# Albedo- and Flavedo-Specific Transcriptome Profiling Related to *Penicillium digitatum* Infection in Citrus Fruit

**DOI:** 10.3390/foods10092196

**Published:** 2021-09-16

**Authors:** María Teresa Lafuente, Paco Romero, Luis González-Candelas

**Affiliations:** Department of Food Biotechnology, Instituto de Agroquímica y Tecnología de Alimentos (IATA-CSIC), Av. Agustín Escardino 7, 46980 Paterna, Valencia, Spain; promero@iata.csic.es (P.R.); lgonzalez@iata.csic.es (L.G.-C.)

**Keywords:** food waste, fungal disease, green mold, peel-tissue specificity, resistance to infection, rot, transcriptomic

## Abstract

*Penicillium digitatum* is the main postharvest pathogen of citrus fruit. Although the inner fruit peel part (albedo) is less resistant than the outer part (flavedo) to *P. digitatum*, the global mechanisms involved in their different susceptibility remain unknown. Here, we examine transcriptome differences between both tissues at fruit harvest and in their early responses to infection. At harvest, not only was secondary metabolism, involving phenylpropanoids, waxes, and terpenoids, generally induced in flavedo vs. albedo, but also energy metabolism, transcription factors (TFs), and biotic stress-related hormones and proteins too. Flavedo-specific induced responses to infection might be regulated in part by ERF1 TF, and are related to structural plant cell wall reinforcement. Other induced responses may be related to H_2_O_2_, the synthesis of phenylpropanoids, and the stress-related proteins required to maintain basal defense responses against virulent pathogens, whereas *P. digitatum* represses some hydrolase-encoding genes that play different functions and auxin-responsive genes in this peel tissue. In infected albedo, the repression of transport and signal transduction prevail, as does the induction of not only the processes related to the synthesis of flavonoids, indole glucosinolates, cutin, and oxylipins, but also the specific genes that elicit plant immunity against pathogens.

## 1. Introduction

Plant and fruit epidermis is constantly exposed to environmental factors, including pathogenic and non-pathogenic microorganisms. Thus, apart from its main function as a protective barrier, the cuticle plays an important role in the interaction with such microorganisms [1]. Colonization of plants and fruit by different microorganisms entails defense responses that aim to limit their growth and development. Whether a host–pathogen interaction is compatible, where disease occurs, or incompatible non-host interactions, where disease does not occur, depend on the rapid efficient deployment of defense responses [2]. The initial defense responses are especially critical for plants’ innate immunity, which is triggered by the recognition of pathogen-associated molecules (PAMs), produced during the interaction with pathogens and recognized by pattern recognition receptors (PRRs) [1,2].

Postharvest fungal pathogens are responsible for much food waste [1]. Contamination of raw materials and products of plant and animal origin with mold and their mycotoxins is currently one of the most important problems faced by food, agricultural, and feed industries around the world. Citrus is one of the major fruit crops in the world. Filamentous necrotrophic fungus known as *Penicillium digitatum* (Pers.:Fr.) Sacc., the causal agent of green mold disease, is the main postharvest pathogen of citrus fruit worldwide. This ascomycete is present on fruit surfaces and penetrates through wounds caused during postharvest handling and storage, which easily lead to rot. The penetration of spores from contaminations in storing and packing houses markedly increases rot incidence, especially if spores are resistant to widely used fungicides. The control of *P. digitatum* is based mostly on applying chemical fungicides. The demand for effective and safe alternative control methods to eliminate mold fungi and their toxic metabolites (mycotoxins) is increasing due to consumer health and environmental concerns and the occurrence of fungicide-resistant strains [3]. Most alternatives rely on biological control microorganisms; chemical or physical treatments, such as ozonation, which is becoming more and more popular [4]; and increasing citrus fruit’s natural defense ability against pathogens [2,3]. Therefore, the study of citrus fruit responses and fungal virulence is drawing increasing interest [5,6,7,8,9].

Transcriptomic studies that focus on either analyzing molecular responses to *P. digitatum* [5,8] or mechanisms related to the elicitation of resistance [10,11,12,13] have been performed in recent years on citrus fruit peel. Yet, despite the inner peel part (albedo) being more susceptible to *P. digitatum* infection than the outer colored part (flavedo) [14], and both the composition and morphology of both peel tissue differing [15,16], no information about the different responses of each specific peel tissue to the pathogen is available. Thus, in relation to the preformed protective fruit barrier against infection caused by phytopathogenic fungi, it is worth noting that the outer peel part of mature citrus fruit is richer than albedo in relevant natural compounds, such as cuticular waxes or phenolic-related compounds [15,16]. Moreover, in line with the present work, it is remarkable that the capability of both peel tissues to provide energy differs [17,18], which is relevant for the elicitation of plant defense responses against pathogens in fruit [13]. Some information also indicates differences between the capability of flavedo and albedo to induce specific defense-related responses against *P. digitatum,* which involves pathogenesis-related (PR) proteins [19] or the enzymatic antioxidant system [14]. Nevertheless, no systematic comprehensive study has been performed to date to unravel the flavedo- and albedo-specific mechanisms putatively involved in their different resistance to *P. digitatum*. To bridge this gap, and to improve knowledge about the molecular mechanisms involved in citrus fruit’s natural defense ability against *P. digitatum*, we transcriptionally compared the molecular mechanisms induced by this fungus in both flavedo and albedo. The initial defense responses are especially relevant for fruit immunity because early response genes are key for perceiving and amplifying stress signals and for inducing downstream gene expression. Therefore, we focused on the early-stage responses produced by *P. digitatum* infection in citrus fruit. We also examined the transcriptome differences of both peel tissues upon fruit harvest before inoculating them with the pathogen. Both approaches revealed complex mechanisms related to citrus fruit’s resistance to *P. digitatum,* which allowed us to distinguish early specific responses of the first (flavedo) and second (albedo) citrus fruit barriers against the pathogen.

## 2. Materials and Methods

### 2.1. Fungal and Fruit Material

In order to infect fruit, *P. digitatum* (Pers.:Fr.) Sacc isolate Pd1 (CECT 20795) [20] was used. The conidial suspension was prepared from 7-day-old cultures grown on Difco^TM^ potato dextrose agar (PDA) (Thermo Fisher Scientific, Wilmington, DE, USA) at 24 °C in sterile distilled water, whose concentration was measured by a hemocytometer as previously described [21]. Finally, the suspension was diluted to 10^4^ conidia mL^−1^ to inoculate fruit.

The mature fruit (a/b external color index 0.47 ± 0.08) of the Navelate (*Citrus sinensis* (L.) Osbeck) orange cultivar were harvested at the end of January (30 January) from adult trees grown in an experimental orchard at the ‘The Spanish Citrus Germplasm Bank’ of the Instituto Valenciano de Investigaciones Agrarias (IVIA) in Moncada (Valencia), Spain. The color index was determined in three replicates of 10 freshly harvested fruit as previously described [22], and was expressed as the a/b Hunter ratio. This ratio, which is classically used for citrus fruit color index determinations, is negative and positive for green and orange fruit, respectively. The a and b values were measured by a Minolta CR-300 Chromameter (Konica Minolta Inc, Ramsey, NJ, USA), and an 8-mm measuring area at three locations around the equatorial plane of each fruit was used [22]. The harvested fruit were immediately delivered to the laboratory, and 225 damage-free oranges were selected.

### 2.2. Fruit Inoculation and Disease Severity Determination

The selected oranges were surface-sterilized with 5% commercial bleach for 5 min and rinsed with tap water as previously described [19]. Then, they were divided into two lots and dried at room temperature for 2 h. The oranges in the first lot, used as the mock wounded control fruit, were inoculated with 10 µL of sterile water. Those in the second lot had the same volume of the 10^4^ conidia mL^−1^ *P. digitatum* suspension. The oranges in each lot were divided into two sublots that contained three replicates of 30 fruit each (first sublot) and three replicates of five fruit each (second sublot). The first sublot was used for the transcriptomic analysis and to periodically analyze changes in the expression levels of the genes selected according to the results obtained from this analysis. For these analyses, peel discs (7 mm in diameter) were taken around the inoculation site and three replicates of 10 fruit per sampling period, and 16 discs per fruit were used in each replicate (160 discs per replicate). To compare any changes in both albedo and flavedo in response to *P. digitatum* inoculation, flavedo and albedo tissues were carefully separated from all the discs with a razor blade, and both tissues were immediately frozen separately and homogenized in liquid nitrogen before being left at −80 °C for the later analyses. The samples inoculated with both the pathogen and sterile water (control) were taken at 1, 2, and 3 d post-inoculation (1, 2, and 3 dpi). Flavedo and albedo discs were also taken from three replicates of 10 fruit from the freshly harvested fruit. The second sublot was made up of three replicates of five fruit and was used to evaluate disease evolution. All the fruit were stored at 20 °C and 90–95% relative humidity (RH) to avoid dehydration.

Fruit were wounded with a flame-sterilized needle at a depth of 4 mm and inoculated with 10 µL of conidial suspension (10^4^ conidia mL^−1^) (infected fruit) or with 10 µL of sterile water (mock-wounded control fruit). Sixteen and four inoculations per fruit were performed on each fruit to take the flavedo and albedo samples for the later analyses and to follow disease severity evolution, respectively.

Disease severity was determined as the mean lesion diameter (mm) of the fruit macerated zone by considering all the inoculated wounds. Diameters were measured in two perpendicular directions on each wound with a flexible ruler before calculating the average diameter of each wound. Three replicate samples, containing five fruit each, with four wounds in the equatorial zone per fruit were used. Lesion diameters were measured periodically in the inoculated fruit left in plastic boxes at 20 °C and 90–95% RH in the dark.

### 2.3. Total RNA Extraction

Total RNA was extracted from 1 g of the flavedo or albedo samples, previously frozen, homogenized, and kept at −80 °C, as previously described [23]. The RNA concentration was determined by a NanoDrop ND-1000 spectrophotometer (Thermo Scientific, Wilmington, DE, USA) and its integrity was verified by migrating 1 µg of RNA on agarose gel [23]. Before the RNA-Seq analysis, RNA quality was assessed by the Agilent 2100 Total RNA Bioanalyzer (Agilent Technologies, Madrid, Spain) using the RNA 6000 Nano Kit (Agilent Technologies, Madrid, Spain).

### 2.4. RNA-Seq, Data Processing and Normalization

Three biological replicates of 2 μg of the RNA of the albedo and flavedo samples from the freshly harvested Navelate oranges, and from the flavedo and albedo samples collected at 1 dpi from the fruit inoculated with *P. digitatum* and their respective mock-wounded control fruit, were employed to construct sequencing libraries, as previously described [23]. They were constructed by means of the TruSeq Stranded mRNA Library Prep Kit^®^ with PolyA selection for ribosomal RNA depletion (Illumina, San Diego, CA, USA) following the manufacturer’s recommendations. The Illumina NextSeq 500 platform was used to sequence libraries and 75-bp single-end reads were generated by the Genome Facility at the SCSIE-UV (Valencia, Spain). The quality of the raw sequence reads was checked by FastP (https://doi.org/10.1093/bioinformatics/bty560, accessed on 30 September 2019) and FastQC v0.11.8 (http://www.bioinformatics.babraham.ac.uk, accessed on 30 September 2019). To obtain clean data, the reads containing only adaptors were removed and sequence reads were filtered by at least a mean Q20. Trimmed sequences were mapped to the *Citrus sinensis* genome (Phytozome release Csinensis_154_v1.1) https://genome.jgi.doe.gov/portal/pages/dynamicOrganismDownload.jsf?organism=Phytozome, accessed on 6 March 2020) with the default settings in the TopHat2 v2.1.0 software. Quality control, visualization, and quantification were performed with the Seqmonk v1.41 software (http://www.bioinformatics.babraham.ac.uk/projects/seqmonk/, accessed on 6 March 2020). Only the reads mapped over protein-coding genes were counted with the RNA-Seq quantification pipeline by assuming opposing strand specificity to generate raw read counts. The edgeR R/Bioconductor package (v3.20.9) in the R (v3.4.4) environment (R Core Team, 2018) was utilized to perform the differential expression analysis between two experimental conditions, which included three biological replicates each. *p*-values were determined by the Benjamini and Hochberg approach, and the genes satisfying an adjusted *p*-value ≤ 0.05 in each comparison were considered differentially expressed genes (DEGs). An estimation of the unique gene expression levels was obtained by the Log_2_ RPM method. Only the DEGs that met a cut-off of |Log_2_ FoldChange| ≥ 1 were considered in the Venn diagrams, which show the number of DEGs induced or repressed by wounding (control) or infection in flavedo and albedo in relation to the same tissue taken at fruit harvest. This cut-off was also established for all the subsequent bioinformatics analyses. For the multivariate analyses, the Seqmonk tool was used to select highly variable genes by a standard deviation cut-off above 0.6. The Log_2_ RPKM values were employed for the principal component analysis (PCA), which was 3D-plotted by plot.ly (https://plot.ly, accessed on 22 June 2020). The selected genes were hierarchically clustered following the average linkage method with Pearson Correlation distance metric, and were then represented by a hierarchical cluster analysis (HCA) and HeatMap according to the MultiExperiment Viewer (MeV 4.9.0). The MapMan software package (http://www.gabipd.de/projects/MapMan/, accessed on 6 December 2020) was used to visualize the gene expression data on a metabolic map. The significantly induced or repressed KEGG metabolic pathways were identified by the KEGG enrichment analysis using the TBtools.KeggBackEnd package (TBtools v 1.046) [24]. The biological processes (BP), molecular functions (MF), and cellular components (CC) enrichment analyses of DEGs were formed by the TopGO correct package [25] with the default ‘weight01′ algorithm. A GO term was considered significantly enriched if more than five DEGs were annotated for that term when the Fisher *p*-value (pgoFisher) was lower than 0.05.

### 2.5. Gene Expression Analysis

For the gene expression analysis, total RNA was treated with ribonuclease-free DNase (Thermo Fisher Scientific, Wilmington, DE, USA) following the manufacturer’s instructions to remove genomic DNA contaminations. A RT-qPCR analysis was carried out following the procedure described in [23] to validate the RNA-Seq results, and to examine the expression pattern of the genes selected in the flavedo and albedo disc samples taken from the mock-wounded (control) fruit and from the fruit inoculated with *P. digitatum*. The cDNA from each sample was synthesized from 2 μg of total RNA by SuperScript III RT (Thermo Fisher Scientific, Wilmington, DE, USA) and ribonuclease inhibitor (Thermo Fisher Scientific, Wilmington, DE, USA), as indicated by the manufacturer. Gene-specific primers were designed with the DNAMAN 4.03 software (Lynnon BioSoft; https://www.lynnon.com/dnaman.html, accessed on 11 January 2021). The forward and reverse primers are summarized in Supplementary Material Table S1. Genes *ACT* and *TUB* were used to normalize the expression levels of the target genes. The expression levels of the flavedo and albedo samples of the control and infected fruit were referred to those obtained in the flavedo and albedo of the freshly harvested fruit, respectively, in the Relative Expression Software Tool (http://rest.gene-quantification.info, accessed on 8 March 2021). Gene-specific primer pairs, and the cDNA obtained from 25 ng of RNA and SYBR Green 1 Master (Roche Diagnostics, Mannheim, Germany), were used to generate the relative gene expression data in a LightCycler480 System (Roche Diagnostics, Mannheim, Germany) instrument. cDNA amplification was monitored during 40 cycles at 95 °C for 10 s, 60 °C for 5 s, and 72 °C for 10 s. Values were the means of three biological replicates samples with two technical replicates ± standard deviation.

### 2.6. Statistical Analysis

Statistical analyses were performed by the STATGRAPHICS software (www.statgraphics.com, accessed on 5 April 2021). A mean comparison by a *t*-test (*p* ≤ 0.05) was made to determine whether the mean gene expression values were significantly different between the infected and mock-wounded control fruit of the same tissue and for the same storage time.

## 3. Results

### 3.1. Disease Severity Evolution in Navelate Fruit Infected by P. digitatum

**Figure 1:**
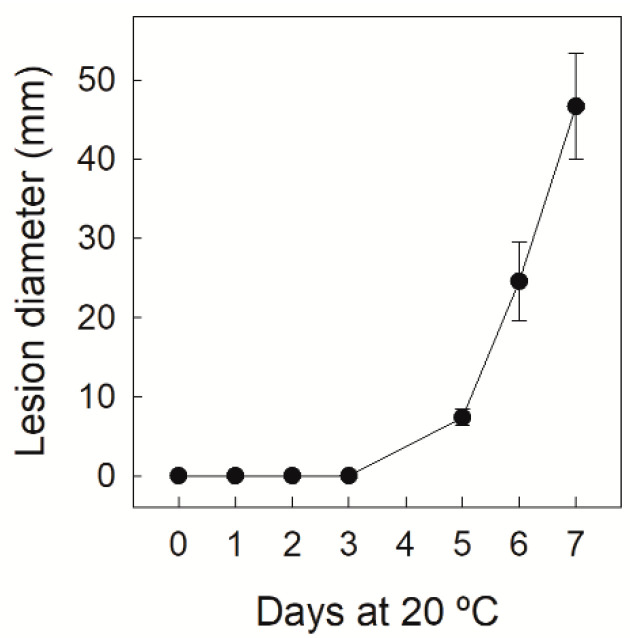
Lesion diameter (mm) of the macerated area of the Navelate orange infected at a depth of 4 mm with 10^4^ conidia mL^−1^ of *P. digitatum* (10 µL). The error interval indicates the standard deviations of the estimated mean value.

Disease severity evolution was evaluated in the Navelate oranges infected by *P. digitatum* (10^4^ conidia mL^−1^) by determining the lesion diameter of the wounds inoculated with the pathogen for up to 7 dpi. As shown in Figure 1, no disease developed in this sweet orange cultivar for up to 3 dpi when this inoculum concentration was applied. It was still low by 5 dpi before sharply increasing until day 7, when about 90% of the inoculated wounds displayed disease symptoms. This percentage was 55% by 5 dpi.

### 3.2. Comparative Transcriptional Profiling during Infection by P. digitatum between Albedo and Flavedo

The transcriptomic differences between outer and inner peel parts were examined at fruit harvest to know the differences in the mechanisms associated with preformed constitutive defense barriers against *P. digitatum* in citrus fruit. Given the relevance of the rapid-signaling transcriptional outputs induced by the host in response to pathogen infection in the host immunity, we examined the very early transcriptional changes that occurred in both peel tissues in response to *P. digitatum* and compared them to those produced during the same period in the mock-wounded tissues. It should be noted that wounding was the control of infection because *P. digitatum* is a wound pathogen.

**Figure 2:**
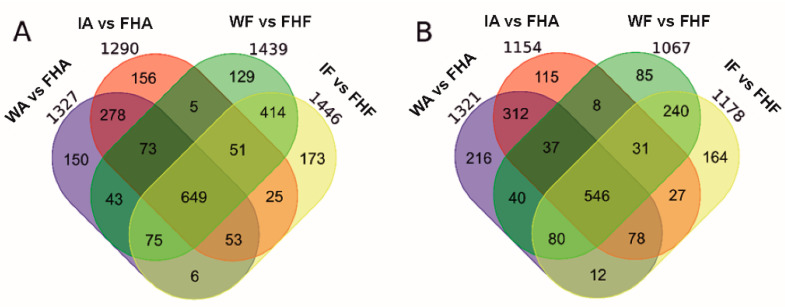
Venn diagrams showing the distribution of the differentially expressed genes (DEG, edgeR, BH *p*-value adjustment α ≤ 0.05) induced (**A**) or repressed (**B**) in the flavedo and albedo tissues of the Navelate oranges inoculated with 10 µL of *P. digitatum* (10^4^ conidia mL^−1^) (I, infected fruit) or 10 µL of water (W, control mock-wounded fruit). After infection or wounding (control of infection), fruit were left in the dark at 20 °C for 1 d (1 dpi). FHF: freshly harvested fruit flavedo; FHA: freshly harvested fruit albedo; WF: wounded flavedo at 1 dpi (control of flavedo infection); WA: wounded albedo at 1 dpi (control of albedo infection); IF: infected flavedo at 1 dpi; IA: Infected albedo at 1 dpi. The expression levels of the up- and down-regulated genes in the wounded (control) and infected fruit were compared to the levels of the freshly harvested fruit, and met a cut-off of |Log_2_ FoldChange| ≥ 1. The numbers outside ellipses are the sum of all the induced (**A**) and repressed (**B**) genes under each particular condition.

The number of up- and down-regulated DEGs at *p* ≤ 0.05 that met a cut-off of at least a two-fold change (−1 ≥ log_2_ ≥ 1) in the wounded and infected albedo and flavedo tissues by 1 dpi, in relation to the same tissue taken at fruit harvest, are summarized in Figure 2. Many genes were up- (Figure 2A) and down-regulated (Figure 2B) by wounding or infection in both peel tissues. The overall number of DEGs was similar when comparing wounding and infection in each tissue (Figure 2). The number of up- and down-regulated DEGs by infection was small compared to wounding (Supplementary Material Table S2). In albedo, 95 DEGs were induced and 35 repressed. The same number of DEGs was repressed in flavedo, and only 19 were up-regulated. The transcriptomic analysis also showed a large number of up- (1604) and down-regulated (1076) DEGs in flavedo in relation to albedo at fruit harvest (Supplementary Material Table S2).

**Figure 3:**
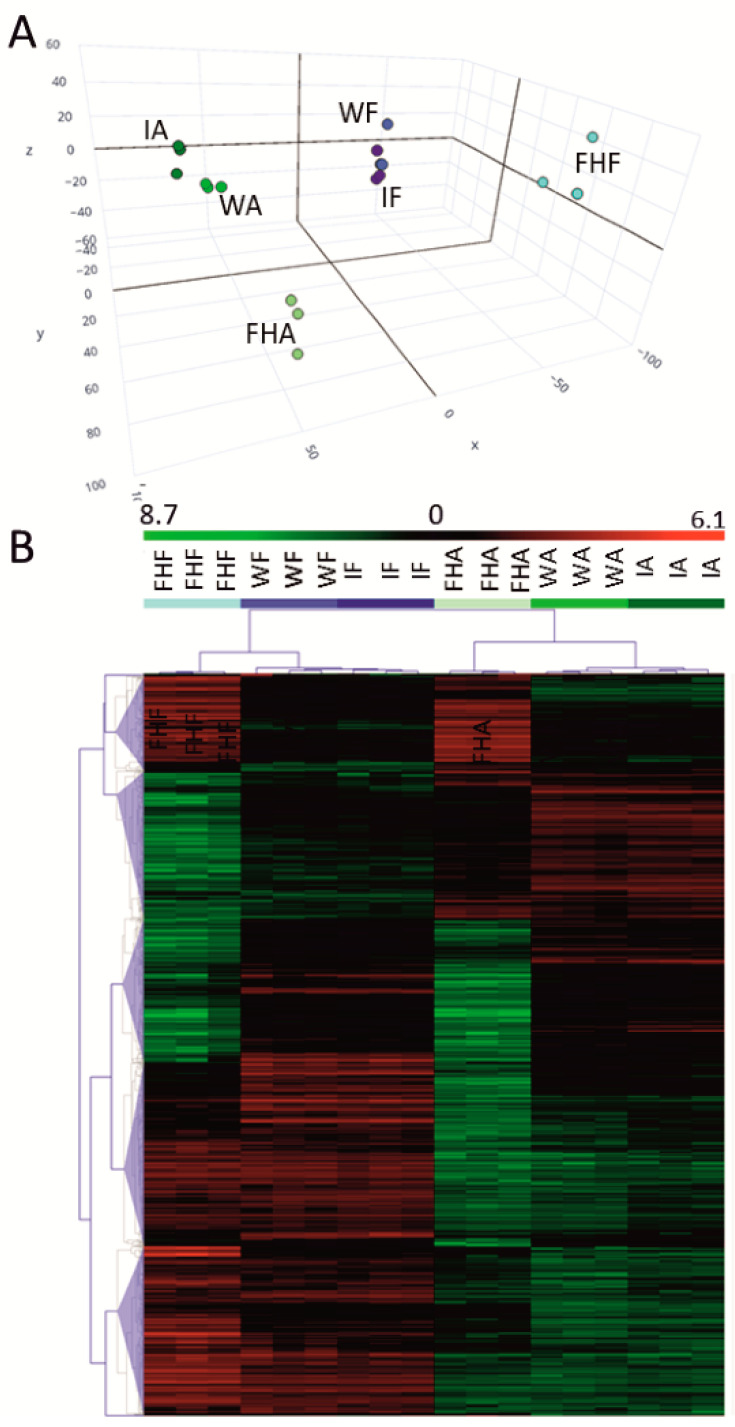
Principal component analysis (PCA) (**A**) and heatmap of the hierarchical cluster analysis (HCA) (**B**) of the expressed genes as determined by the RNA-Seq analysis. All the genes were considered in the PCA (A), whereas the HCA was based on those DEGs meeting a cut-off of STDEV > 0.6 and |Log_2_ FoldChange| ≥ 1 for all the represented conditions: freshly harvested fruit flavedo (FHF); freshly harvested fruit albedo (FHA); wounded flavedo at 1 dpi (WF); wounded albedo at 1 dpi (WA); infected flavedo at 1 dpi (IF); infected albedo at 1 dpi (IA). The colors in the HCA for each condition are consistent with those in the PCA. Heatmap colors vary from light green (repression) to dark red (induction) on a Log_2_ scale. Three biological replicates from each condition were used for the analyses.

These results coincide with results of the PCA and HCA analyses. The PCA results (Figure 3A) revealed that the transcriptional profiles of the three biological replicates of each sample were mostly grouped together, which indicates good repeatability of the RNA-Seq data across replications. The analysis also revealed marked differences in the gene expression pattern between albedo and flavedo at harvest, and also between wounded and/or infected tissues, compared to the same tissue from the freshly harvested fruit. Differences between the infected and wounded control tissues for the same peel tissue were less relevant. This trend also agreed with the gene clustering shown in the heatmap analysis according to the gene expression profiles. For this analysis, 1153 DEGs were identified in all the comparisons as they only met a standard deviation cut-off above 0.6. They were grouped into five clusters (Figure 3B), which showed marked differences between peel tissues, and in response to wounding or infection in each tissue, but with negligible differences between the infected and wounded samples of the same peel tissue. The consistency and sensitivity of the RNA-Seq analysis were validated by RT-qPCR after determining the expression of the selected genes (Supplementary Material Table S1) because a correlation close to 0.9 (r^2^ = 0.863 *p* ≤ 0.05) was found between both analyses (Supplementary Material Figure S1). Therefore, the RNA-Seq results were reliable for further analyses.

### 3.3. Comparison of the Induced or Repressed Metabolic Pathways in Albedo in Relation to Flavedo in Navelate Orange at Fruit Harvest

The identification of the differential metabolic pathways between the flavedo and albedo of the freshly harvested Navelate oranges was performed by a KEGG analysis (Table 1). The pathways induced in albedo vs. flavedo involved starch and sucrose metabolism and signal transduction, which mostly included the DEGs belonging to the ‘plant hormone signal transduction’ pathway related to auxin synthesis and regulation, and also to auxin-induced proteins (Supplementary Material Table S3) [26,27]. This tissue was also enriched in the cellular processes involving transporters. The metabolic pathways induced in flavedo vs. albedo (down-regulated in albedo; Table 1) belonged to energy metabolism and the secondary metabolism, which involved cutin, suberin and wax biosynthesis, diverse terpenoids, stilbenoids, as well as the biosynthesis of flavonoids, with a much lower *p*-value (7.83 × 10^−8^) than the other pathway subcategories (Table 1).

**Figure 4:**
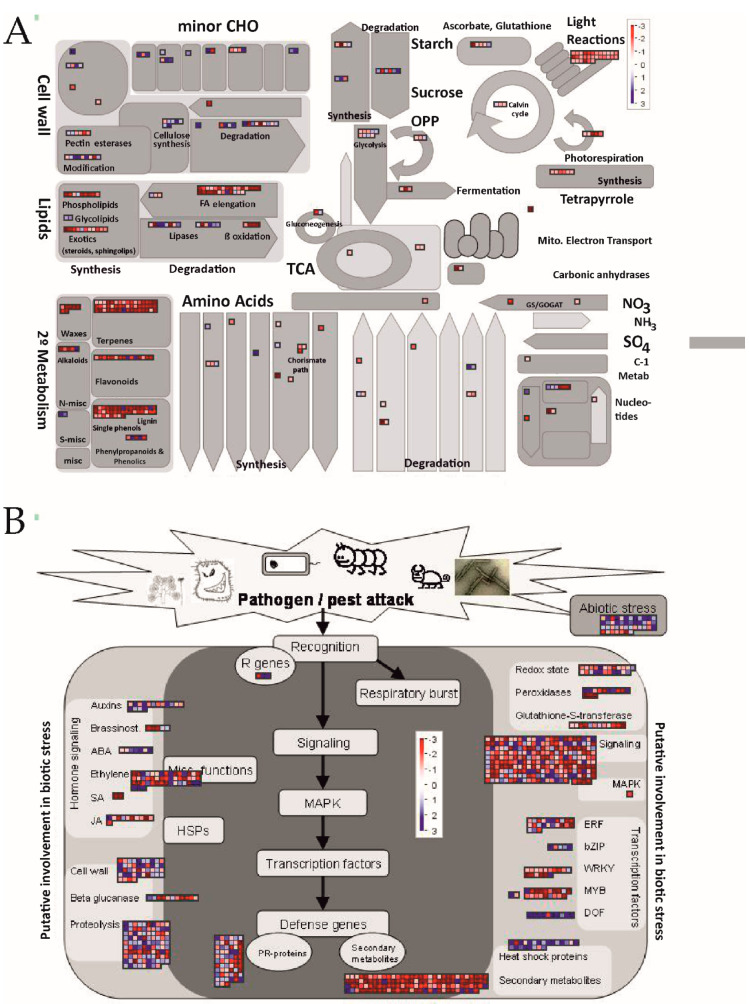
Metabolic (**A**) and biotic stress (**B**) overviews using MapMan to compare the transcript accumulation in the albedo of the freshly harvested fruit vs. the flavedo of the samples taken from the peel of the same fruit. Red and blue squares represent DEGs with decreasing and increasing transcript levels in albedo vs. flavedo. Only the DEGs that met a cut-off of |Log_2_ FoldChange| ≥ 1 were included in the analysis. The color scale, ranging from −3 to 3, is indicated in the figure and is expressed as a log_2_fold change.

Fatty acid elongation and degradation were induced in the metabolism of lipids, as was the α-linoleic metabolism. The metabolic pathways related to the glutathione metabolism and the degradation of different amino acids were induced in this tissue. The examination of BRITE hierarchies further showed relevant differences in the expression of the transcription factors (TFs) between both tissues and highlighted the repression of photosynthesis in albedo, which showed the lowest corrected *p*-value (4.40 × 10^−12^) (Table 1). The visualization of the gene expression data on the general metabolic overview map (Figure 4A) agreed with the KEGG analysis (Table 1). The examination of the different specific metabolic pathways showed major changes in the biotic stress-related responses (Figure 4B) involving secondary metabolism, proteolysis, cell wall, and the pathogenesis-related (PR) proteins and abundant genes involved in redox status, which were generally more repressed in albedo. Relevant differences between both tissues were also found in the expression of the genes playing a signaling role, including hormone signaling, encoding abundant receptor-like protein kinase (RLKs) and the TFs belonging to different families. The overall results indicate that these transcripts were generally less expressed in albedo, but this trend was reversed when examining DOFs TFs, a family of plant-specific TFs of the zinc finger superfamily containing a highly conserved DNA-binding motif called the Dof domain (Figure 4A).

### 3.4. Albedo and Flavedo Early Responses to P. digitatum Infection in Citrus Fruit

The examination of the DEGs with an ortholog in Arabidopsis (Supplementary Material Table S4) indicated that, in both peel tissues, most of the DEGs regulated by *P. digitatum* were related to defense stress-related responses in plants. Some DEGs were commonly induced by infection in both tissues, while others were specific to each peel part. The functional categorization of these DEGs identified that different BP, MF, and CC were significantly repressed or induced in an early infection stage (Table 2). Of the induced processes in albedo (Table 2), MFs’ ‘terpene synthase activity’ and ‘protein dimerization activity’, which mostly grouped the O-methyltransferases (OMTs)-encoding genes (Supplementary Material Table S5), included the DEGs that were exclusive of these processes. However, a common set of genes was identified in the other induced processes. Of these, the process containing the largest number of DEGs was ‘oxidation–reduction’ BP. Most of the DEGs in this category also belonged to the iron ion and hemebinding MFs, which grouped the same genes and encoded different CYP450 proteins participating in the synthesis of secondary metabolites, such as amino acid derivatives (CYP706A4 and CYP82C4), flavonoids (3 CYP75B1) and indole glucosinolates (2 CYP83B1 and 1 CYP86B) (http://www-ibmp.u-strasbg.fr/~CYPedia, accessed on 25 June 2021) (Supplementary Material Table S5). It is worth stressing the major increases in the expression of not only ethylene responsive factors (ERF1), nodulins, or the plant stearoyl-acyl-carrier protein desaturases (SACPD), but also of other genes associated with plant defense against pathogens, such as crinkly4 (ACR4) or AGD2-like defense response protein (ALD1) (Supplementary Material Table S4). Of the repressed processes in albedo, different processes contained a subset of the DEGs identified among those included in ‘oxidation–reduction’ BP (Supplementary Material Table S5). They were the ‘flavin adenine dinucleotide binding’ MF and ‘oxidoreductase activity’, as well as the ‘iron ion binding’ and ‘heme binding’ MFs, which contained almost the same DEGs. The genes in these subsets mostly encoded different CYP450 proteins related to the catabolism of hormones gibberellic acid (GA) and abscisic acid (ABA) (CYP707A4 and CYP94C3), or the metabolism of cell wall carbohydrates (CYP76G1) and glutathione (CYP71B37). The other differential repressed processes contained exclusive DEGs, except the ‘steroid biosynthetic process’ BP and the ‘3-β-hydroxy-δ5-steroid dehydrogenase’ MF. The DEGs in the other processes participate in signal transduction or transport, affect the tonoplast, or encode a major facilitator superfamily protein (MFSP) and the P-glycoprotein 11 (Pgp-11) involved in membrane transport.

In flavedo, the induction of the processes related to the chloroplast/light stimulus prevailed, which included a chloroplast signal recognition particle component (CAO), and cell wall biosynthesis involving a cellulose synthase (CSLB4) and carboxypeptidase activity (Table 2 and Supplementary Material Table S5). The DEGs included in these processes were among the most induced by infection, in relation to wounding, in flavedo, together with a glyoxal oxidase (GLOX)-related protein and some OMTs, TFs, and a RLK1 (Supplementary Material Table S4). Of the repressed processes (Table 2), the fungus had a major effect on the processes involving hydrolase activity, which affected glucan metabolism or other proteins such as Pgp-11, a subtilase, and two purple acid phosphatases (PAPs) (Supplementary Material Table S5). Infection also repressed processes related to the response to auxin and the biosynthesis of glycolipids (Table 2). In this tissue, the cell wall and apoplast CC, which included the same DEGs (Supplementary Material Table S5), were also repressed (Table 2).

Only one BP and two MF processes were commonly regulated in both peel tissues, which involved the repression of the RmlC-like cupin and the CXXS1-encoding genes in both tissues, as well as the induction of the different DEGs that encode OMT proteins, which differed between albedo and flavedo (Table 2, Supplementary Material Table S5). The ‘biotic stress overview’ examination after the MapMan analysis (Supplementary Material Figure S2) further highlighted that *P. digitatum* infection had a stronger impact on the induction of the biotic-stress related responses in albedo than in flavedo. Major differences were found in the DEGs involved in signaling, which mostly encoded the RLKs belonging to different families. This analysis also showed a stronger effect of infection on the metabolic changes related to secondary metabolism, cell wall modification, or the involved PRs, and also on the expression of two TFs (WRKY72 and AP2/EREBP) in albedo.

Changes in the expression of the DEGs selected for the RT-qPCR validation of RNA-Seq were analyzed for up to 3 dpi (Supplementary Material Figure S3) before disease symptoms developed. The results indicated that most DEGs responded to both wounding and infection, which agrees with the RNA-Seq analysis data (Supplementary Material Table S2). In some cases, the differences between the control (wounded) and infected samples from the same tissue were transient and did not remain after 1 dpi, while these differences increased in other cases. The differences in the expression of some genes between the control and infected samples in one peel tissue by day 1 occurred later in the other peel tissue.

## 4. Discussion

The control of green mold disease caused by *P. digitatum* is based mostly on using fungicides. The development of alternative control methods would benefit if our knowledge about the mechanisms involved in citrus fruit’s natural defense ability against this fungus increased. As flavedo is less susceptible than albedo to infection [14], here we compared the: (1) transcriptome differences between flavedo and albedo at fruit harvest to better understand the molecular mechanisms involved in natural constitutive defense barriers against fungal invasion; and (2) early fruit responses to *P. digitatum* attack of each peel tissue before disease symptoms develop (Figure 1).

As expected, an examination of the transcriptome differences between albedo and flavedo at fruit harvest showed the induction of both cuticular waxes and phenylpropanoids, which are involved in natural or elicited resistance against *P. digitatum* in citrus fruit [5,10,18,19,28,29,30] in flavedo *versus* albedo (Table 1, Figure 4A). This result agreed with the induction of energy metabolism, which is relevant for both the synthesis of secondary metabolites and the elicitation of resistance against *P. digitatum* in citrus fruit [13]. Moreover, favoring the cell energetics in citrus fruit peel induces plant defense responses against pathogens [31]. The expression of the abundant genes involved in photosynthesis and related to light reactions was also higher in flavedo (Figure 4A). Sesquiterpenoid, triterpenoid, and terpenoid–quinone biosynthesis was also induced in flavedo. Although monoterpenes may stimulate *P. digitatum* infection [32], many terpenoids protect plants from pathogen attack [33]. Moreover in citrus fruit, terpenoid metabolism has been linked with elicited resistance [13]. Lipid metabolism, which involves lipids related to the cuticle, and also to fatty acid degradation, lipid β-oxidation, and α-linolenic acid metabolism, which are relevant for jasmonates biosynthesis, was also induced (Table 1, Figure 4). This coincides with the fact that most jasmonate-related DEGs were less expressed in albedo at fruit harvest (Figure 4B). Among the plant hormones, it was noteworthy that most ethylene-related genes were more expressed in flavedo (Figure 4B). As both hormones play a defensive role against *P. digitatum* in citrus fruit [5,34], they should be key components in the first preformed constitutive defense barrier of this crop. It was surprising to find that hormonal signal transduction was repressed in flavedo, but most of the DEGs on this pathway were related to auxins (Supplementary Material Table S3) [26,27]. As auxins are important for cell wall expansion [27] and albedo cells are considerably larger than those of flavedo [15], auxin-related differences between both peel parts might be explained by their different morphology. Nevertheless, we should bear in mind that, as auxins affect the induction of cell wall-related enzymes [27], we cannot rule out their participation in the *P. digitatum*–citrus fruit interaction. The results presented herein also highlight the greater relevance of not only the ERF TFs, which may be key regulators of symptomatic *versus* asymptomatic signaling during necrotrophic fungal colonization [35], but also of the WRKY and MYB transcriptional regulators in flavedo (Figure 4B). The up-regulation of different TFs during the elicitation of resistance against *P. digitatum* has been shown [12], but their function in citrus fruit immunity against *P. digitatum* is still practically unknown. However, a recent report has shown that the overexpression of *CsWRKY65*, which was more expressed in flavedo at fruit harvest, favors ROS accumulation and *PR* gene expression [36]. RLKs and other stress-related genes involved in plant defense against pathogens [1], which might protect the outer peel part of the ROS secreted by *P. digitatum* [6] (e.g., glutathione-S-transferase or peroxidase), glucanases, and other PRs, previously related to citrus fruit resistance against *P. digitatum* [10,14,19,30], were also more expressed generally in flavedo (Figure 4B). Therefore, constitutive defense against *P. digitatum* in the outer peel part was not only limited to secondary metabolism, and involving phenylpropanoids, waxes, and terpenoids, but also to energy metabolism, TFs, signaling molecules, and the biotic stress-related proteins involving plant hormones, PRs, and ROS homeostasis.

Considerably fewer DEGs were regulated by infection than the DEGs found when comparing albedo and flavedo at fruit harvest (Figure 2 and Figure 3). No early specific responses of albedo and flavedo to *P. digitatum* infection have been reported. An earlier study examined the transcriptomic changes induced by *P. digitatum* in the Jincheng 447^#^ citrus cultivar [8]. However, that study was conducted in the pericarp, did not discriminate flavedo and albedo tissues, used a much higher concentration of the conidial suspension (10^6^ rather than 10^4^), and its RNA-Seq analysis was performed later after inoculation. Therefore, the present study bridges the knowledge gap about the specific responses of inner and outer peel parts to the pathogen, and is more restrictive when examining early responses to infection. In fact, under our experimental conditions, the number of up- and down-regulated genes in either the albedo or flavedo of the infected fruit in relation to the control wounded fruit was much smaller (<150 up- + down-regulated in each peel tissue) than those found previously in the pericarp (4353 up-/4724 down-regulated) [8]. Some DEGs were up-regulated by infection in both pericarp tissues (Supplementary Material Table S4). Of them, the two ERF1 TFs were remarkable because ERF1 has been related to eliciting resistance against *P. digitatum* [29]. Likewise, it is worth noting the induction of the receptor kinase RLK1, which is a key PRRs component for the recognition of PAMPs, and the cysteine protease RD21 for being a crucial component of plant immunity [37]. A pectin methylesterase (PME) inhibitor, Pgp-11, which is an ABC-type xenobiotic transporter, a CXXS1 thioredoxin, and an RmlC-like cupin were all repressed in both peel tissues (Supplementary Material Table S4). An RmlC-like cupin has been described as a pathogenicity factor in fungi. RmlC-like cupins may regulate host defenses [38], but no information about the role of plant RmlC-like cupins and their ability to cope with pathogens has yet provided. In contrast, it is known that ABC transporters, and the control of both ROS and cell wall-degrading enzymes secreted by phytopathogenic fungi, are important host mechanisms to counteract toxic compounds and pathogen virulence [1,6]. As *P. digitatum* secretes PME and ROS-related responses to colonize citrus fruit [6,9], the repressions of these genes in both tissues could reflect a fungal mechanism to reduce citrus fruit resistance to infection.

By focusing on early specific flavedo-induced responses to infection, the obtained results unveiled the relevance of both the chloroplast, which is necessary for photosynthesis and is one of the main sources of ROS production [1], and of the cell wall (Table 2, Supplementary Material Table S5), in the resistance of flavedo against *P. digitatum*. In line with this, it is worth noting the results showing major increases in the expression of two genes (CAO and GLOX) encoding H_2_O_2_-generating enzymes in the flavedo of the infected fruit compared to wounding (Supplementary Material Table S4), which suggests flavedo’s better ability to favor ROS in response to this pathogen. Regarding the cell wall, the up-regulation of CSLB4, involved in cellulose synthesis, should be a flavedo defense response against *P. digitatum,* which secretes cellulases to infect citrus fruit [9]. It is also worth mentioning that, besides maintaining cell wall integrity, cellulose can mediate plant defense responses against pathogens [39]. However, hemicellulose synthesis seemed to be down-regulated in flavedo in response to infection (Supplementary Material Table S5). Specific responses to infection in flavedo can also suggest the involvement of lignification in the defense of the outer peel part against *P. digitatum*, which would be in concordance with previous findings showing that immature citrus fruit produce higher lignin content and are more resistant than commercial and overmature fruit to *P. digitatum* infection [40]. It is noteworthy that lignification increased in citrus fruit peel following *P. digitatum* infection [41], and our results revealed marked increases in the expression levels of two OMT1s (orange1.1 g 043449 m.g and orange1.1 g 046424 m.g; Supplementary Material Table S4) in flavedo, which were homologous to an Arabidopsis caffeate O-methyltransferase relevant for lignin biosynthesis in plants. Some of the above-mentioned DEGs were more prominently expressed in the infected than in the wounded albedo, but the increases in the expression levels in this tissue were generally lower or not statistically significant. Therefore, it would appear that flavedo’s ability to favor lignification in response to *P. digitatum* was greater than that of the inner peel part. As plant OMTs constitute a large family of enzymes that may also lead to the synthesis of different alkaloids and phenylpropanoids, further research should be conducted to clarify whether up-regulated OMTs are involved in lignification and/or whether they participate in the synthesis of other phenylpropanoid derivatives displaying antifungal activity against *P. digitatum*. We also found the induction of the ERF1 TFs, which may up-regulate lignin biosynthetic genes in plants [42], and we observed major expression levels of the genes encoding H_2_O_2_-generating enzymes CAO and GLOX in the infected flavedo vs. its respective control (wounded flavedo). H_2_O_2_ may play a role in attacking *P. digitatum* [43,44] and in activating defense genes in plants [45], but could also make the cell wall stronger against microbial enzyme attack by favoring lignification [45,46].

The up-regulation in both tissues of cytochrome CYP706A4 is also remarkable (Supplementary Material Table S4), which is responsible for the biosynthesis of amino acids and downstream derivatives, as is the CYP75B1 flavonoid 3′-hydroxylase, which participates in the elicited resistance against this pathogen [10,12,16]. Lastly, the most specific gene induced in flavedo with a 17.9-fold increase was a ZIP TF showing homology to an Arabidopsis gene involved in callose metabolism regulation, which further suggests the relevance of cell wall reinforcement in flavedo to cope with *P. digitatum* infection. The transcriptomic analysis also highlighted the repression of the ‘hydrolase activity’ MF in the *P. digitatum*–flavedo interaction (Table 2, Supplementary Material Table S5), which included a set of genes encoding XHT hydrolases, GDSL-like lipase/Acylhydrolase, subtilase, PAP proteins, and a calcineurin-like metallo-phosphoesterase (MPE), whose domain is found in PAPs. These proteins may be required for maintaining basal defense responses against virulent pathogens in plants [47,48,49] and, therefore, the repression of these genes could be related to an early virulence mechanism induced by *P. digitatum* to favor flavedo colonization. In line with this, it is worth noting that, although XTHs may function as primary wall-loosening agents, they might also favor xyloglucan depolymerization and the release of xyloglucan oligosaccharides, which can initiate defense signaling and, hence, trigger plant immunity [50]. The ‘response to auxins’ was also repressed. As auxins are a prime plant defense against necrotrophic fungal pathogen infection [51], and have been related to resistance elicitation against *P. digitatum* in citrus fruit [29], it would seem that auxins repression in flavedo may also play a role in the *P. digitatum*–flavedo compatible interaction.

The results presented herein also revealed that defense responses in the inner peel part against *P. digitatum* are less likely to be related to cell wall reinforcement than in the outer peel part. They also revealed that the metabolic pathways favoring the synthesis of terpenoids and oxidation–reduction processes mostly related to CYP450-encoding proteins (Table 2, Supplementary material Table S5), and involved in pest resistance and participating in the synthesis of amino acids derivatives, flavonoids, and indole glucosinolates, which may possess antifungal activity [51,52,53], may operate in albedo. This result agrees with the induction of the ‘protein dimerization activity’ MF, which includes OMTs and a bHLH-encoding gene that may participate in the regulation of flavonoid and glucosinolate pathways [42]. The DEGs in the oxidoreduction process also encoded CYP450 proteins involved in acyl–lipid metabolism, which participate in both wax and cutin (CYP86B1) [54], and in oxylipin metabolism (CYP71B10) (http://www-ibmp.u-strasbg.fr/~CYPedia, accessed on 25 June 2021). In line with this, it is remarkable that a stearoyl-acyl carrier protein desaturase, which favors jasmonic acid-mediated pathways [55], was also up-regulated, and that *P. digitatum* mycelial growth can be promoted by citrus fruit epicuticular wax and the conidial germination inhibited by cutin [56]. Thus, it would seem that, in response to infection, albedo redirects the metabolism toward the synthesis of the defensive compounds that are already constitutively present in flavedo. An examination of the other DEGs highly induced by infection in albedo (Supplementary Material Table S4) also revealed the relevance of defense response proteins such as the phospholipase C, which has been previously related to citrus fruit defense against *P. digitatum* [57], the ALD1, required for the biosynthesis of pipecolic acid, which activates local and long-distance defense signaling [58], and of proteins acting as PRs in immunity (ACR4) [59] or playing a role in intercellular communication such as ENOD 15 (11.5-fold change). Although nodulin-like activities at the plant–microbe interface may be important for pathogens to enhance their fitness during host colonization, ENOD 15 is activated by plant immunity elicitors [60]. As a β–glucosidase-encoding DEG was also highly induced by infection in albedo vs. wounding (20.3-fold change), we cannot rule out the notion that the release of aglycones is a mechanism that operates in albedo to activate the defense response against pathogen attack [61]. Inoculation with the pathogen also led to the repression of oxidation–reduction processes because of the repression of the DEGs involved in the catabolism of GA and ABA, in the metabolism of cell wall carbohydrate or in glutathione (Supplementary Material Table S5). These results, together with the repression of peroxidase, indicate that GA and ABA may act in early infection stages in albedo, and suggest that preventing the ROS-dependent defense of albedo is a factor that contributes to *P. digitatum* virulence. These ideas not only agree with a previous study that identified the genes involved in the virulence of this pathogen toward citrus fruit [6], but also with the results showing that ABA plays a protective role against *P. digitatum* in citrus fruit [21]. Other repressed processes in albedo also suggest that *P. digitatum* may operate in the inner peel part by negatively affecting signal transduction and transport.

## 5. Conclusions

Taken together, the herein presented data suggest that constitutive defenses against *P. digitatum* in the flavedo of freshly harvested fruit involve: (1) energy metabolism; (2) secondary metabolism, including phenylpropanoids, lipids, waxes and terpenoids; (3) ERF, WRKY, and MYB TFs; and (4) signaling molecules, and biotic stress-related proteins involving plant hormones, PRs, and ROS homeostasis, which were generally less expressed in the albedo. The early specific induced responses against *P. digitatum* infection in flavedo might be regulated by ERF1 TF and are mostly associated with the reinforcement of the plant cell wall. Other protective-related responses might be related to either H_2_O_2_ or the synthesis of phenylpropanoids, while the repression of both hydrolase-encoding genes and auxin-responsive genes would contribute to *P. digitatum* virulence and colonization in this outer peel tissue. In contrast, the induction of the secondary metabolism-related processes, involving terpenoids, phenylpropanoids and oxylipins; the synthesis of wax and cutin-related compounds; and the repression of signal transduction and transport, prevailed in albedo.

## Figures and Tables

**Table 1:** Metabolic pathways and BRITE hierarchies were identified as induced (UP) or repressed (DOWN) by KEGG analysis in the albedo of the freshly harvested Navelate fruit in relation to the flavedo samples taken from the same peel samples. Three biological replicates from each condition were used. Only the DEGs (*p* ≤ 0.05) showing at least a 2-fold change in expression were included in the analysis.

	Pathway/Brite Map	Term Name	Enrich Factor	Corrected *p*-Value (BH Method)
		Pathway		
	A09100	Metabolism		
	00500	Starch and sucrose metabolism	2.84	3.52 × 10^−2^
	A09130	Environmental Information Processing	2.54	1.04 × 10^−4^
UP	B 09132	Signal transduction	2.44	4.17 × 10^−4^
	04075	Plant hormone signal transduction	2.53	1.52 × 10^−3^
	A09180	Brite Hierarchies		
		Protein families: signaling and cellular processes		
	02000	Transporters	1.95	6.21 × 10^−3^
		Pathway		
	A09100	Metabolism	1.97	1.24 × 10^−13^
	B 09101	Carbohydrate metabolism	1.57	1.71 × 10^−3^
	00640	Propanoate metabolism	4.30	1.38 × 10^−3^
	B 09102	Energy metabolism	2.14	2.31 × 10^−4^
	00195	Photosynthesis	5.87	1.43 × 10^−7^
	00196	Photosynthesis—antenna proteins	8.73	1.07 × 10^−5^
	B 09103	Lipid metabolism	2.59	1.46 × 10^−9^
	00062	Fatty acid elongation	4.21	2.45 × 10^−3^
	00071	Fatty acid degradation	4.13	9.09 × 10^−4^
	00073	Cutin, suberine and wax biosynthesis	5.82	3.69 × 10^−4^
	00592	alpha-Linolenic acid metabolism	3.55	2.34 × 10^−3^
	B 09105	Amino acid metabolism	1.55	2.26 × 10^−2^
	00280	Valine, leucine and isoleucine degradation	3.12	9.77 × 10^−3^
DOWN	B 09106	Metabolism of other amino acids	2.19	1.32 × 10^−3^
	00480	Glutathione metabolism	2.58	7.50 × 10^−3^
	B 09109	Metabolism of terpenoids and polyketides	3.26	1.25 × 10^−9^
	00900	Terpenoid backbone biosynthesis	3.50	1.73 × 10^−3^
	00909	Sesquiterpenoid and triterpenoid bios…	4.58	7.35 × 10^−3^
	00900	Terpenoid backbone biosynthesis	3.50	1.73 × 10^−3^
	B 09110	Biosynthesis of other secondary metabolites	3.04	1.26 × 10^−10^
	00940	Phenylpropanoid biosynthesis	3.27	8.81 × 10^−8^
	00945	Stilbenoid, diarylheptanoid and gingerol bios.	7.64	5.69 × 10^−4^
	00941	Flavonoid biosynthesis	6.68	7.83 × 10^−8^
	A09180	Brite Hierarchies		
	B 09181	Protein families: metabolism	1.47	1.65 × 10^−4^
	00194	Photosynthesis proteins	6.51	4.40 × 10^−12^
	00199	Cytochrome P450	5.73	1.08 × 10^−8^
	01006	Prenyltransferases	3.67	7.00 × 10^−4^
	01004	Lpid biosynthesis proteins	2.96	2.29 × 10^−3^
		Pathway		
	A09100	Metabolism		
	00904	Diterpenoid biosynthesis	5.52 up	3.27 × 10^−2^
UP/			4.28 down	4.95 × 10^−3^
DOWN	A09180	Brite Hierarchies		
		Protein families: genetic information processing		
	03000	Transcription factors	2.25 up	4.87 × 10^−3^
			1.82 down	2.34 × 10^−3^

**Table 2:** Gene ontology (GO) analysis (*p* ≤ 0.05) of the biological (BP) and molecular function (MF) processes and of the cell components (CC) induced (↑) or repressed (↓) in flavedo (F) and/or albedo (A) by 1 d post-inoculation (1 dpi) of the Navelate fruit inoculated with 10 μL of a 10^4^ conidia mL^−1^ suspension of *P. digitatum* spores in relation to their wounded (control) samples. Three biological replicates from each condition were used. Only the DEGs (*p* ≤ 0.05) showing at least a 2-fold change in expression were included in the analysis.

GO Category	GO ID	Term	Up		Down	
Pattern 1: Regulated by *P. digitatum* in the flavedo						
BP	GO:0045038	protein import into chloroplast thylakoi...	↑	1.30 × 10^−3^		
BP	GO:0009416	response to light stimulus	↑	1.47 × 10^−2^		
BP	GO:0030244	cellulose biosynthetic process	↑	3.45 × 10^−2^		
MF	GO:0016760	cellulose synthase (UDP-forming) activit...	↑	2.21 × 10^−2^		
MF	GO:0004185	serine-type carboxypeptidase activity	↑	4.27 × 10^−2^		
CC	GO:0080085	signal recognition particle, chloroplast...	↑	6.70 × 10^−4^		
CC	GO:0009507	chloroplast	↑	4.69 × 10^−3^		
BP	GO:0006073	cellular glucan metabolic process			↓	2.10 × 10^−3^
BP	GO:0009733	response to auxin			↓	2.60 × 10^−3^
BP	GO:0009247	glycolipid biosynthetic process			↓	2.51 × 10^−2^
MF	GO:0016762	xyloglucan:xyloglucosyl transferase acti...			↓	5.30 × 10^−4^
MF	GO:0016787	hydrolase activity			↓	1.17 × 10^−3^
CC	GO:0048046	apoplast			↓	2.40 × 10^−4^
CC	GO:0005618	cell wall			↓	2.31 × 10^−3^
Pattern 2: Regulated by *P. digitatum* in the albedo						
BP	GO:0055114	oxidation-reduction process	↑	9.20 × 10^−5^	↓	2.40 × 10^−3^
BP	GO:0006631	fatty acid metabolic process	↑	4.00 × 10^−2^		
MF	GO:0016705	oxidoreductase activity, acting on paire...	↑	4.50 × 10^−7^	↓	1.61 × 10^−2^
MF	GO:0005506	iron ion binding	↑	1.10 × 10^−6^	↓	1.71 × 10^−2^
MF	GO:0020037	heme binding	↑	4.60 × 10^−6^	↓	3.40 × 10^−3^
MF	GO:0045300	acyl-[acyl-carrier-protein] desaturase a...	↑	5.40 × 10^−4^		
MF	GO:0046983	protein dimerization activity	↑	9.90 × 10^−3^		
MF	GO:0010333	terpene synthase activity	↑	4.53 × 10^−2^		
CC	None					
BP	GO:0000160	phosphorelay signal transduction system			↓	3.10 × 10^−3^
BP	GO:0006694	steroid biosynthetic process			↓	4.85 × 10^−2^
MF	GO:0005215	transporter activity			↓	2.20 × 10^−3^
MF	GO:0050660	flavin adenine dinucleotide binding			↓	8.90 × 10^−3^
MF	GO:0003854	3-beta-hydroxy-delta5-steroid dehydrogen...			↓	3.84 × 10^−2^
CC	None					
Pattern 3: Commonly regulated by *P. digitatum* in the albedo and flavedo						
BP	GO:0006662	glycerol ether metabolic process			↓A	2.72 × 10^−2^
BP	GO:0006662	glycerol ether metabolic process			↓F	4.50 × 10^−2^
MF	GO:0008171	O-methyltransferase activity	↑A	3.50 × 10^−3^		
MF	GO:0008171	O-methyltransferase activity	↑F	8.05 × 10^−3^		
MF	GO:0045735	nutrient reservoir activity			↓A	1.28 × 10^−2^
MF	GO:0045735	nutrient reservoir activity			↓F	1.62 × 10^−2^
CC	None					

## Data Availability

Data supporting the findings of this study are available in this publication and its Supplementary Materials published online. The datasets used for the transcriptomic study can be found at the NCBI repository (BioProject ID PRJNA749665). https://dataview.ncbi.nlm.nih.gov/object/PRJNA749665?reviewer=hqrs4dtug289ml6f51ndcgbisf, created on 26 July 2021.

## Supplementary Materials

The following are available online at https://www.mdpi.com/article/10.3390/foods10092196/s1, Table S1: The selected genes and primers used for the RT-qPCR analysis; Table S2: Global RNA-Seq analysis data; Table S3: List of the DEGs in the ‘Hormonal signal transduction’ pathway with a higher expression in albedo than in flavedo at fruit harvest; Table S4: List of the DEGs (*p*-adjusted value ≤ 0.05) under the conditions indicated in each sheet meeting a cut-off of |Log_2_ Fold Change| ≥ 1. Values are the fold change expression (FC) and Log_2_ FC of the compared conditions. Positive numbers mean higher expression values in the first term of the comparison, while negative values denote higher expression values in the second term; Table S5: Identification of the DEGs included in each induced or repressed biological process (BP), molecular function (MF) and cellular component (see Table 1) in the albedo and flavedo of the infected Navelate oranges in relation to their control wounded samples; Figure S1: Multiple linear regression analysis (R2) for the comparisons made between the RNA-Seq and the RT-qPCR gene expression data for the DEGs listed in Supplementary Table S1. The expression of these DEGs was quantified in the albedo and flavedo of the freshly harvested fruit and in the wounded and infected albedo and flavedo peel tissues taken from Navelate oranges at 1 dpi; Figure S2: Biotic stress overview using MapMan to compare the transcript accumulation in the albedo (A) and flavedo (B) of the Navelate oranges inoculated with *P. digitatum* (10^4^ conidia mL^−1^) in relation to the flavedo- and albedo-wounded control samples inoculated with water, respectively. Red and blue squares represent the DEGs with decreasing and increasing transcript levels in the infected tissues vs. wounded tissues. Only the DEGs meeting a cut-off of |Log_2_ FoldChange| ≥1 were included in the analysis. The color scale is indicated in the figure and is expressed as log_2_ fold change; Figure S3: changes in the relative gene expression of the selected DEGs in albedo (▲,△) and flavedo (●,○) of the Navelate oranges inoculated with *P. digitatum* (10^4^ conidia mL^−1^) (▲,●) or water (wounded control samples, △,○) in relation to the albedo and flavedo of the freshly harvested fruit, respectively. After infection, fruit were left in the dark at 20 °C. The error interval indicates the standard deviation of the estimated mean value. * denotes significant differences (*p* ≤ 0.05) between the infected and wounded flavedo for the same storage time according to the *t*-test. For albedo, ** was used rather than *.

## References

[1] Alkan N., Fortes A.M. (2015). Insights into molecular and metabolic events associated with fruit response to post-harvest fungal pathogens. Front. Plant Sci..

[2] Tian S., Torres R., Ballester A.-R., Li B., Vilanova L., González-Candelas L. (2016). Molecular aspects in pathogen-fruit interactions: Virulence and resistance. Postharvest Biol. Technol..

[3] Palou L., Ali A., Fallik E., Romanazzi G. (2016). GRAS, plant- and animal-derived compounds as alternatives to conventional fungicides for the control of postharvest diseases of fresh horticultural produce. Postharvest Biol. Technol..

[4] Ozkana R., Smilanickb J.L., Karabuluta O.A. (2011). Toxicity of ozone gas to conidia of *Penicillium digitatum*, *Penicillium italicum*, and *Botrytis cinerea* and control of gray mold on table grapes. Postharvest Biol. Technol..

[5] González-Candelas L., Alamar S., Sánchez-Torres P., Zacarías L., Marcos J.F. (2010). A transcriptomic approach highlights induction of secondary metabolism in citrus fruit in response to *Penicillium digitatum* infection. BMC Plant Biol..

[6] López-Pérez M., Ballester A.-R., González-Candelas L. (2015). Identification and functional analysis of *Penicillium digitatum* genes putatively involved in virulence towards citrus fruit. Mol. Plant Pathol..

[7] OuYang Q., Tao N., Guoxing J. (2016). Transcriptional profiling analysis of *Penicillium digitatum*, the causal agent of citrus green mold, unravels an inhibited ergosterol biosynthesis pathway in response to citral. BMC Genom..

[8] Deng B., Wang W., Deng L., Yao S., Ming J., Zeng K. (2018). Comparative RNA-seq analysis of citrus fruit in response to infection with three major postharvest fungi. Postharvest Biol. Technol..

[9] Qian X., Yang Q., Zhang Q., Abdelhai M.H., Dhanasekaran S., Serwah B.N.A., Gu N., Zhang H. (2019). Elucidation of the initial growth process and the infection mechanism of *Penicillium digitatum* on postharvest citrus (*Citrus reticulata* Blanco). Microorganisms.

[10] Ballester A.-R., Lafuente M.T., Forment J., Gadea J., de Vos R.C.H., Bovy A.G., González-Candelas L. (2011). Transcriptomic profiling of citrus fruit peel tissues reveals fundamental effects of phenylpropanoids and ethylene on induced resistance. Mol. Plant Pathol..

[11] Hershkovitz V., Sela N., Taha-Salaime L., Liu J., Rafael G., Kessler C., Aly R., Levy M., Wisniewski M., Droby S. (2013). De-novo assembly and characterization of the transcriptome of *Metschnikowia fructicola* reveals differences in gene expression following interaction with *Penicillium digitatum* and grapefruit peel. BMC Genom..

[12] Chen O., Deng L., Ruan C., Yi L., Zeng K. (2021). *Pichia galeiformis* induces resistance in postharvest citrus by activating the phenylpropanoid biosynthesis pathway. J. Agric. Food Chem..

[13] Lafuente M.T., Romero P., Ballester A.-R. (2021). Coordinated activation of the metabolic pathways induced by LED blue light in citrus fruit. Food Chem..

[14] Ballester A.R., Lafuente M.T., González-Candelas L. (2006). Spatial study of antioxidant enzymes, peroxidase and phenylalanine ammonia-lyase in the citrus fruit-*Penicillium digitatum* interaction. Postharvest Biol. Technol..

[15] Cajuste J.F., García-Breijo F.J., Reig-Armiñana J., Lafuente M.T. (2011). Ultrastructural and histochemical analysis reveals ethylene-induced responses underlying reduced peel collapse in detached citrus fruit. Microsc. Res. Tech..

[16] Ballester A.-R., Teresa Lafuente M., González-Candelas L. (2013). Citrus phenylpropanoids and defence against pathogens. Part II: Gene expression and metabolite accumulation in the response of fruits to *Penicillium digitatum* infection. Food Chem..

[17] Establés-Ortiz B., Romero P., Ballester A.-R., González-Candelas L., Lafuente M.T. (2016). Inhibiting ethylene perception with 1-methylcyclopropene triggers molecular responses aimed to cope with cell toxicity and increased respiration in citrus fruits. Plant Physiol. Biochem..

[18] Romero P., Alférez F., Lafuente M.T. (2020). Involvement of phospholipases and sucrose in carbon starvation-induced non-chilling peel pitting in citrus fruit. Postharvest Biol. Technol..

[19] Ballester A.-R., Izquierdo A., Lafuente M.T., González-Candelas L. (2010). Biochemical and molecular characterization of induced resistance against *Penicillium digitatum* in citrus fruit. Postharvest Biol. Technol..

[20] Marcet-Houben M., Ballester A.-R., de la Fuente B., Harries E., Marcos J.F., González-Candelas L., Gabaldón T. (2012). Genome sequence of the necrotrophic fungus *Penicillium digitatum*, the main postharvest pathogen of citrus. BMC Genom..

[21] Lafuente M.T., Ballester A.-R., González-Candelas L. (2019). Involvement of abscisic acid in the resistance of citrus fruit to *Penicillium digitatum* infection. Postharvest Biol. Technol..

[22] Lafuente M.T., Alférez F., Romero P. (2014). Postharvest ethylene conditioning as a tool to reduce quality loss of stored mature sweet oranges. Postharvest Biol. Technol..

[23] Romero P., Lafuente M.T. (2020). Abscisic acid deficiency alters epicuticular wax metabolism and morphology that leads to increased cuticle permeability during sweet orange (*Citrus sinensis*) Fruit Ripening. Front. Plant Sci..

[24] Chen C., Chen H., Zhang Y., Thomas H.R., Frank M.H., He Y., Xia R. (2020). TBtools: An integrative toolkit developed for interactive analyses of big biological data. Mol. Plant.

[25] Alexa A., Rahnenfuhrer J., Lengauer T. (2006). Improved scoring of functional groups from gene expression data by decorrelating GO graph structure. Bioinformatics.

[26] Weiste C., Dröge-Laser W. (2014). The *Arabidopsis* transcription factor bZIP11 activates auxin-mediated transcription by recruiting the histone acetylation machinery. Nat. Commun..

[27] Majda M., Robert S. (2018). The role of auxin in cell wall expansion. Int. J. Mol. Sci..

[28] Cajuste J.F., González-Candelas L., Veyrat A., García-Breijo F.J., Reig-Armiñana J., Lafuente M.T. (2010). Epicuticular wax content and morphology as related to ethylene and storage performance of “Navelate” orange fruit. Postharvest Biol. Technol..

[29] Lu L., Wang J., Zhu R., Lu H., Zheng X., Yu T. (2015). Transcript profiling analysis of *Rhodosporidium paludigenum*-mediated signalling pathways and defense responses in mandarin orange. Food Chem..

[30] Youssef K., Sanzani S.M., Ligorio A., Ippolito A., Terry L.A. (2014). Sodium carbonate and bicarbonate treatments induce resistance to postharvest green mould on citrus fruit. Postharvest Biol. Technol..

[31] Romero P., Alférez F., Establés-Ortiz B., Lafuente M.T. (2020). Insights into the regulation of molecular mechanisms involved in energy shortage in detached citrus fruit. Sci. Rep..

[32] Droby S., Eick A., Macarisin D., Cohen L., Rafael G., Stange R., McColum G., Dudai N., Nasser A., Wisniewski M. (2008). Role of citrus volatiles in host recognition, germination and growth of *Penicillium digitatum* and *Penicillium italicum*. Postharvest Biol. Technol..

[33] Singh B., Sharma R.A. (2015). Plant terpenes: Defense responses, phylogenetic analysis, regulation and clinical applications. 3 Biotech.

[34] Droby S., Porat R., Cohen L., Weiss B., Shapiro B., Philosoph-Hadas S., Meir S. (1999). Suppressing green mold decay in grapefruit with postharvest jasmonate application. J. Am. Soc. Hortic. Sci..

[35] Baetsen-Young A., Chen H., Shiu S.H., Day B. (2021). Contrasting transcriptional responses to *Fusarium virguliforme* colonization in symptomatic and asymptomatic hosts. Plant Cell.

[36] Wang W., Li T., Chen Q., Deng B., Deng L., Zeng K. (2021). Transcription factor CsWRKY65 participates in the establishment of disease resistance of citrus fruits to *Penicillium digitatum*. J. Agric. Food Chem..

[37] Balakireva A.V., Zamyatnin A.A. (2018). Indispensable role of proteases in plant innate immunity. Int. J. Mol. Sci..

[38] El Hadrami A., Islam M.R., Adam L.R., Daayf F. (2015). A cupin domain-containing protein with a quercetinase activity (VdQase) regulates *Verticillium dahliae’s* pathogenicity and contributes to counteracting host defenses. Front. Plant Sci..

[39] Sasidharan R., Voesenek L.A., Pierik R. (2011). Cell wall modifying proteins mediate plant acclimatization to biotic and abiotic stresses. CRC Crit. Rev. Plant Sci..

[40] Vilanova L., Torres R., Viñas I., González-Candelas L., Usall J., Fiori S., Solsona C., Teixidó N. (2013). Wound response in orange as a resistance mechanism against *Penicillium digitatum* (pathogen) and *P. expansum* (non-host pathogen). Postharvest Biol. Technol..

[41] Vilanova L., Viñas I., Torres R., Usall J., Jauset A.M., Teixidó N. (2012). Infection capacities in the orange-pathogen relationship: Compatible (*Penicillium digitatum*) and incompatible (*Penicillium expansum*) interactions. Food Microbiol..

[42] Meraj T.A., Fu J., Raza M.A., Zhu C., Shen Q., Xu D., Wang Q. (2020). Transcriptional factors regulate plant stress responses through mediating secondary metabolism. Genes.

[43] Macarisin D., Cohen L., Eick A., Rafael G., Belausov E., Wisniewski M., Droby S. (2007). Postharvest pathology and mycotoxins *Penicillium digitatum* suppresses production of hydrogen peroxide in host tissue during infection of citrus fruit. Phytopathology.

[44] Buron-Moles G., Torres R., Teixidó N., Usall J., Vilanova L., Viñas I. (2015). Characterisation of H_2_O_2_ production to study compatible and non-host pathogen interactions in orange and apple fruit at different maturity stages. Postharvest Biol. Technol..

[45] Klenell M., Morita S., Tiemblo-Olmo M., Mühlenbock P., Karpinski S., Karpinska B. (2005). Involvement of the chloroplast signal recognition particle cpSRP43 in acclimation to conditions promoting photooxidative stress in Arabidopsis. Plant Cell Physiol..

[46] Daou M., Faulds C.B. (2017). Glyoxal oxidases: Their nature and properties. World J. Microbiol. Biotechnol..

[47] Ravichandran S., Stone S.L., Benkel B., Prithiviraj B. (2013). *Purple Acid Phosphatase5* is required for maintaining basal resistance against *Pseudomonas syringae* in *Arabidopsis*. BMC Plant Biol..

[48] Figueiredo J., Silva M.S., Figueiredo A. (2018). Subtilisin-like proteases in plant defence: The past, the present and beyond. Mol. Plant Pathol..

[49] Su H.G., Zhang X.H., Wang T.T., Wei W.L., Wang Y.X., Chen J., Zhou Y.B., Chen M., Ma Y.Z., Xu Z.S. (2020). Genome-wide identification, evolution, and expression of GDSL-type esterase/lipase gene family in soybeag. Front. Plant Sci..

[50] Claverie J., Balacey S., Lemaître-Guillier C., Brulé D., Chiltz A., Granet L., Noirot E., Daire X., Darblade B., Héloir M.-C. (2018). The cell wall-derived xyloglucan is a new DAMP triggering plant immunity in Vitis vinifera and *Arabidopsis thaliana*. Front. Plant Sci..

[51] Qi L., Yan J., Li Y., Jiang H., Sun J., Chen Q., Li H., Chu J., Yan C., Sun X. (2012). *Arabidopsis thaliana* plants differentially modulate auxin biosynthesis and transport during defense responses to the necrotrophic pathogen *Alternaria brassicicola*. New Phytol..

[52] Noordermeer M.A., Veldink G.A., Vliegenthart J.F.G. (2001). Fatty acid hydroperoxide lyase: A plant cytochrome P450 enzyme involved in wound healing and pest resistance. ChemBioChem.

[53] Smolen G., Bender J. (2002). Arabidopsis cytochrome P450 *cyp83B1* mutations activate the tryptophan biosynthetic pathway. Genetics.

[54] Compagnon V., Diehl P., Benveniste I., Meyer D., Schaller H., Schreiber L., Franke R., Pinot F. (2009). CYP86B1 is required for very long chain ω-hydroxyacid and α,ω-dicarboxylic acid synthesis in root and seed suberin polyester. Plant Physiol..

[55] Kachroo A., Shanklin J., Whittle E., Lapchyk L., Hildebrand D., Kachroo P. (2006). The Arabidopsis stearoyl-acyl carrier protein-desaturase family and the contribution of leaf isoforms to oleic acid synthesis. Plant Mol. Biol..

[56] Ding S., Zhang J., Yang L., Wang X., Fu F., Wang R., Zhang Q., Shan Y. (2020). Changes in cuticle components and morphology of ‘Satsuma’ mandarin (*Citrus unshiu*) during ambient storage and their potential role on *Penicillium digitatum* infection. Molecules.

[57] Lafuente M.T., Ballester A.-R., Holland N., Cerveró J., Romero P. (2021). Interrelation between ABA and phospholipases D, C and A_2_ in early responses of citrus fruit to *Penicillium digitatum* infection. Postharvest Biol. Technol..

[58] Vlot A.C. (2021). A quest for long-distance signals: The epidermis as central regulator of pipecolic acid-associated systemic acquired resistance. J. Exp. Bot..

[59] Stahl Y., Faulkner C. (2016). Receptor complex mediated regulation of symplastic traffic. Trends Plant Sci..

[60] Denancé N., Szurek B., Noël L.D. (2014). Emerging functions of nodulin-like proteins in non-nodulating plant species. Plant Cell Physiol..

[61] Le Roy J., Huss B., Creach A., Hawkins S., Neutelings G. (2016). Glycosylation is a major regulator of phenylpropanoid availability and biological activity in plants. Front. Plant Sci..

