# Supplementary material for: Albedo- and Flavedo-Specific Transcriptome Profiling Related to Penicillium digitatum Infection in Citrus Fruit
Source: Foods. 2021 Sep 16;10(9):2196. doi: 10.3390/foods10092196 (PMC8467057; doi:10.3390/foods10092196)

Table S1. The selected genes and primers used for the RT-qPCR analysis

Gene	Citrus ID	Description	Forward (5' - 3')	Reverse (5' - 3')
CsALD1	orange1.1g013521m.g	AGD2-like defense response protein 1	GACCGTGATTGACT ACTACA	TGCACTGACTCTGATAT GTT
CsSACPD	orange1.1g037659m.g	Plant stearoyl-acyl-carrier-protein desaturase family protein	TGTTACCATTACTCA AACCA	GTCTCATCTCTGACACC ATC
CsERF1	orange1.1g039409m.g	ethylene response factor 1	CAGAAATAAGGGAT TCAACT	GAATGTTTCCTCTTTAG TGC
CsCAO	orange1.1g016433m.g	chloroplast signal recognition particle component (CAO)	TCTCAATTTCTTCAC ATCAA	CATTCGATTAGGTACTC CAT
CsGLOX	orange1.1g048017m	glyoxal oxidase-related protein	TGTTACCTTCTGGTG ATGTA	TACTCCCACCCACTAAT ATC
CsCSLB04	orange1.1g038469m.g	cellulose synthase-like B4	TCAGACTACATTCGT ATTGG	CATTTTATCATCGTCTT CG
CsPAP8	orange1.1g026017m.g	purple acid phosphatase 8	TCAATATCTACACTG CTCCA	AATCATAAACATGGTC TTCC
CsSAUR	orange1.1g046487m.g	SAUR-like auxin-responsive protein family	CCATTAGAAAGTCA AGCAA	GAGACAAGAAAGAGA TTGGA
CsACT	orange1.1g037845m.g	Actin	TTAACCCCAAGGCC AACAGA	TCCCTCATAGATTGGTA CAGTATGAGA
CsTUB	orange1.1g036688m.g	Tubulin	GCATCTTGAACCCG GTAC	ATCAATTCGGCGCCTTC AG

Table S2. Global RNA-Seq analysis data (See Excel file)

Table S3. List of the DEGS in the 'Hormonal signal transduction' pathway with a higher expression in albedo than in flavedo at fruit harvest

Orange ID	Description	Symbol	log2FoldChange	padj
orange1.1g044130m.g	putative indole-3-acetic acid-amido synthetase GH3.9	GH3.9	6.022	8.55×10^{-20}
orange1.1g022618m.g	phytochrome-associated protein 2	IAA27,PAP2	4.521	2.8×10^{-21}
orange1.1g046192m.g	response regulator 9	ARR9,ATRR4	4.2	9.74×10^{-3}
orange1.1g026247m.g	response regulator 9	ARR9,ATRR4	4.188	2.17×10^{-18}
orange1.1g031731m.g	histidine-containing phosphotransmitter 1	AHP1	3.864	7.39×10^{-17}
orange1.1g001846m.g	CHASE domain containing histidine kinase protein	AHK4,ATCRE1,CRE1,WOL,WOL1	3.804	2.76×10^{-45}
orange1.1g038233m.g	bZIP transcription factor family protein	bZIP65,TGA10	3.259	1.26×10^{-34}
orange1.1g045443m.g	xyloglucan endotransglucosylase/hydrolase 16	XTH16	2.791	4.41×10^{-40}
orange1.1g029186m.g	SAUR-like auxin-responsive protein family	0	2.649	2.81×10^{-27}
orange1.1g007728m.g	Auxin-responsive GH3 family protein	GH3.1	2.626	2.18×10^{-20}
orange1.1g020697m.g	ABA-responsive element binding protein 3	AREB3,DPBF3	2.546	5.60×10^{-9}
orange1.1g022632m.g	auxin-induced protein 13	IAA13	2.393	7.79×10^{-25}
orange1.1g034523m.g	SAUR-like auxin-responsive protein family	0	2.326	5.59×10^{-5}
orange1.1g029511m.g	indoleacetic acid-induced protein 16	IAA16	2.175	5.94×10^{-7}
orange1.1g026239m.g	response regulator 9	ARR9,ATRR4	1.917	6.25×10^{-7}
orange1.1g046390m.g	SAUR-like auxin-responsive protein family	0	1.583	2.30×10^{-3}
orange1.1g036039m.g	bZIP transcription factor family protein	bZIP21,TGA9	1.513	3.70×10^{-9}
orange1.1g011392m.g	Transmembrane amino acid transporter family protein	AUX1,MAP1,PIR1,WAV5	1.399	2.08×10^{-14}
orange1.1g022696m.g	xyloglucan endotransglucosylase/hydrolase 5	EXGT-A4,XTH5	1.302	1.71×10^{-18}
orange1.1g048660m.g	response regulator 6	ARR6	1.271	1.67×10^{-4}
orange1.1g043131m.g	GRAS family transcription factor family protein	RGA,RGA1	1.191	1.23×10^{-8}
orange1.1g044419m.g	SAUR-like auxin-responsive protein family	0	1.057	3.00×10^{-3}
orange1.1g043077m.g	histidine-containing phosphotransfer factor 5	AHP5	1.019	1.39×10^{-6}

Table S4. List of the DEGs (p-adjusted value ≤ 0.05) under the conditions indicated in each sheet meeting a cut-off of |Log_2 Fold Change| ≥ 1. Values are the fold change expression (FC) and Log_2 FC of the compared conditions. Positive numbers mean higher expression values in the first term of the comparison, while negative values denote higher expression values in the second term (See Excel file)

Table S5. Identification of the DEGs included in each induced or repressed biological process (BP), molecular function (MF) and cellular component (CC) (see Table 1) in the albedo and flavedo of the infected Navelate oranges in relation to their control wounded samples (See Excel file)

Figure S1. Multiple linear regression analysis (R2) for the comparisons made between the RNA-Seq and the RT-qPCR gene expression data for the DEGs listed in Supplementary Table S1. The expression of these DEGs was quantified in the albedo and flavedo of the freshly harvested fruit and in the wounded and infected albedo and flavedo peel tissues taken from Navelate oranges at 1 dpi

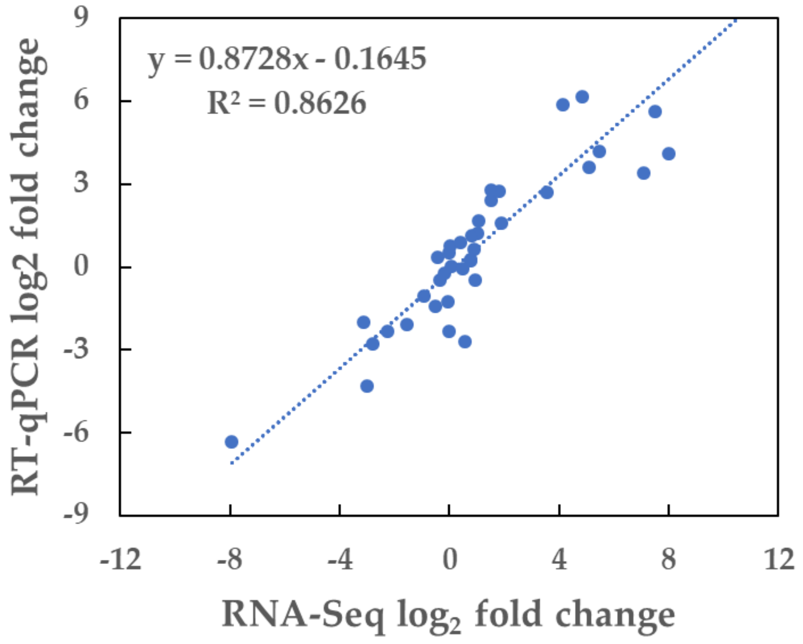

Figure S2. Biotic stress overview using MapMan to compare the transcript accumulation in the albedo (A) and flavedo (B) of the Navelate oranges inoculated with *P. digitatum* (10^4 conidia mL^{-1}) in relation to the flavedo- and albedo-wounded control samples inoculated with water, respectively. Red and blue squares represent the DEGs with decreasing and increasing transcript levels in the infected tissues vs. wounded tissues. Only the DEGs meeting a cutoff of |Log2 FoldChange| ≥1 were included in the analysis. The color scale is indicated in the figure and is expressed as $\log_2$ fold change

A

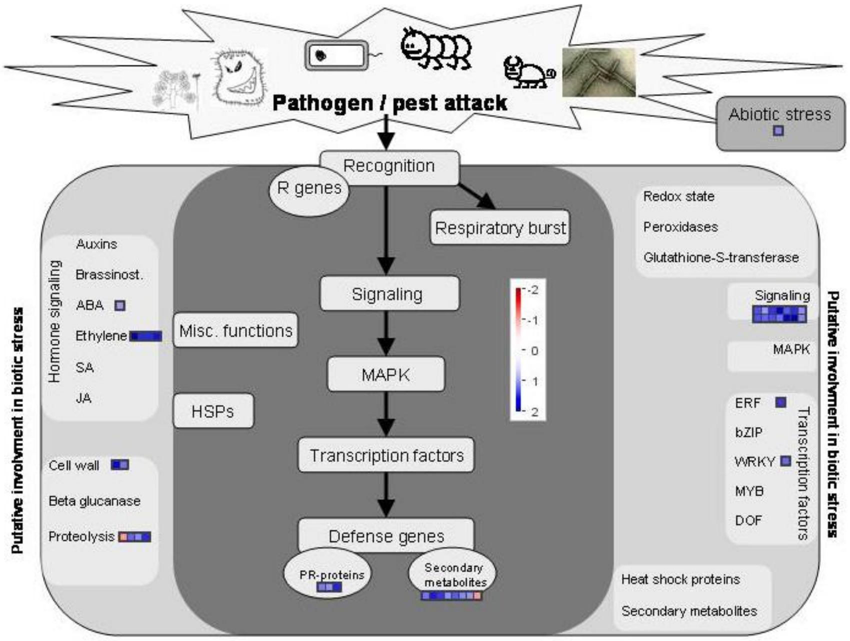

B

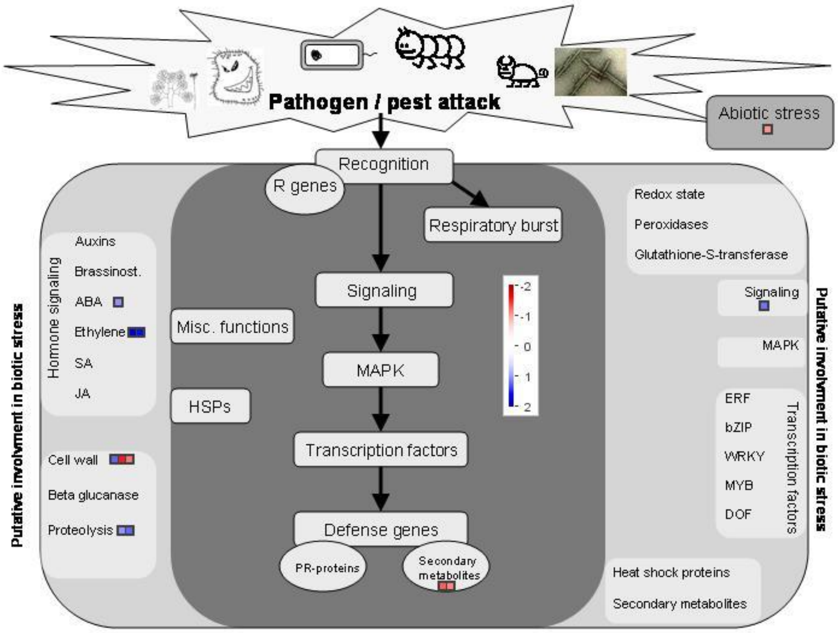

Supplementary Material Figure S3. Changes in the relative gene expression of the selected DEGS in albedo (▲,△) and flavedo (●,○) of the Navelate oranges inoculated with *P. digitatum* (10^4 conidia mL^{-1}) (▲,●) or water (wounded control samples, △,○) in relation to the albedo and flavedo of the freshly harvested fruit, respectively. After infection, fruit were left in the dark at 20 ºC. The selected DEGs were: A) *CsERF1*; B) *CsCAO*; C) *CsGLOX*; D) *CsCSLB04*; E) *CsSACPD*; F) *CsALD1*; G) *CsSAUR*; H) *CsPAP8*. The error interval indicates the standard deviation of the estimated mean value. * denotes significant differences ($p \leq 0.05$) between the infected and wounded flavedo for the same storage time according to the *t*-test. For albedo, ** was used rather than *.

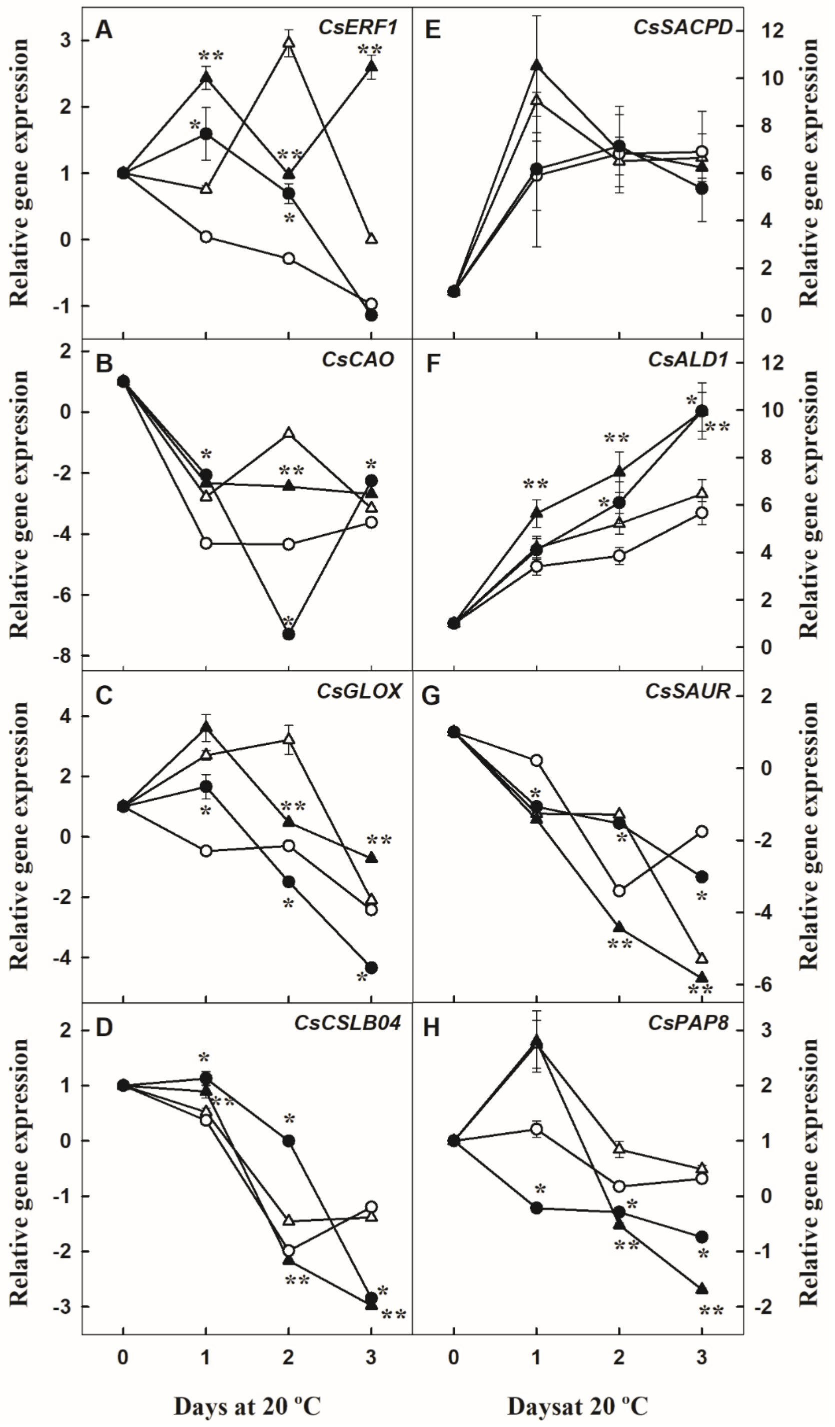

Supplement: Supplementary file 1 [file foods-10-02196-s001.zip › Proof_Lafuente_et al_Supplementary Material- Foods-1338235-revised.pdf]
